# Four Weeks Treatment with Glecaprevir/Pibrentasvir + Ribavirin—A Randomized Controlled Clinical Trial

**DOI:** 10.3390/v14030614

**Published:** 2022-03-16

**Authors:** Lone W. Madsen, Peer B. Christensen, Janne F. Hansen, Birgit T. Røge, Dorte K. Holm, Sandra Dröse, Anne Øvrehus

**Affiliations:** 1 Department of Infectious Diseases, Odense University Hospital, 5000 Odense, Denmark; peer.christensen@rsyd.dk (P.B.C.); janne.fuglsang.hansen@rsyd.dk (J.F.H.); sandra.droese@rsyd.dk (S.D.); anne.oevrehus@rsyd.dk (A.Ø.); 2 OPEN, Odense Patient Data Explorative Network, Odense University Hospital, 5000 Odense, Denmark; 3 Clinical Institute, University of Southern Denmark, 5000 Odense, Denmark; 4 Unit for Infectious Diseases, Department of Medicine, Sygehus Lillebælt, 6000 Kolding, Denmark; birgit.thorup.roege@rsyd.dk; 5 Department of Clinical Immunology, Odense University Hospital, 5000 Odense, Denmark; dorte.holm@rsyd.dk

**Keywords:** chronic hepatitis C, HCV, DAA, glecaprevir, pibrentasvir, ribavirin, predictors, genotype, viral load

## Abstract

Enhancing treatment uptake for hepatitis C to achieve the elimination goals set by the World Health Organization could be achieved by reducing the treatment duration. The aim of this study was to compare the sustained virological response at week 12 (SVR12) after four weeks of glecaprevir/pibrentasvir (GLE/PIB) + ribavirin compared to eight weeks of GLE/PIB and to estimate predictors for SVR12 with four weeks of treatment through a multicenter open label randomized controlled trial. Patients were randomized 2:1 (4 weeks:8 weeks) and stratified by genotype 3 and were treatment naïve of all genotypes and without significant liver fibrosis. A total of 27 patients were analyzed for predictors for SVR12, including 15 from the first pilot phase of the study. In the ‘modified intention to treat’ group, 100% (7/7) achieved cure after eight weeks and for patients treated for four weeks the SVR12 was 58.3% (7/12). However, patients with a baseline viral load <2 mill IU/mL had 93% SVR12. The study closed prematurely due to the low number of included patients due to the COVID-19 pandemic. Our results suggest that viral load should be taken into account when considering trials of short course treatment.

## 1. Introduction

The estimated global prevalence of chronic hepatitis C is 56.8 million infected individuals, and around 275,000 people die annually of chronic liver disease and liver cancer because of untreated hepatitis C infection [1]. In the absence of a vaccine, treatment with highly active direct-acting antivirals (DAA) has led to the strategy of treatment for chronic hepatitis C as being prevention [2]. This has also been reflected in the World Health Organization’s (WHO) elimination goals, aiming for a 90% reduction in the incidence of hepatitis C virus (HCV) by year 2030 [3]. However, the path to elimination is not necessarily easy and achievable by year 2030. Even for high-income countries, recent data have shown that many countries are off track to reach elimination within a decade [4,5] and improvement across the cascade of care is needed to achieve the ambitious goals [6].

The high cost of DAA has been a major obstacle for treatment uptake. The introduction of generic products in low and middle income countries (LMICs) has improved this significantly [7], but differences in treatment prices between countries still persist [8].

Reducing treatment duration will not only benefit the overall cost, but also adherence. The prevalence of HCV infection among people who inject drugs (PWID) is substantial higher than the general population [9], and in this population adherence can be a major issue for treatment uptake. Previously studies have identified that adherence to DAA declined persistently during the treatment course [10,11].

So far, the clinical trials of ultra-short treatment of four weeks or less have overall been disappointing and characterized by a low number of participants [12,13,14,15,16,17,18]. The 4RIBC study was designed in two phases where the aim of the first phase was to evaluate if four weeks’ treatment with glecaprevir/pibrentasvir (GLE/PIB) without ribavirin would be feasible [19]. It showed a cure rate of 73% after four weeks’ treatment with GLE/PIB + ribavirin, and 59% without ribavirin. The challenge for ultra-short duration, highlighted by WHO, is to find the target population that can be cured with DAA in a shorter duration than current guidelines recommend [20]. Based on the results of phase 1, the aim of the second phase of the 4RIBC trial was to identify the target population for short treatment duration.

The objective was to compare the percentage of subjects achieving SVR12 after four weeks of treatment with GLE/PIB + ribavirin compared to eight weeks of treatment with GLE/PIB. Adverse events and safety parameters were observed up to the expected SVR12 point and hepatitis C RNA was monitored before and after treatment. Furthermore, we investigated predictors for achieving cure after ultra-short treatment for Hepatitis C, including data analysis from both phases of the 4RIBC study.

## 2. Materials and Methods

### 2.1. Study Design

We performed an investigator-initiated multicenter randomized, open label clinical trial (EudraCT No. 2017-005179-21).

Patients were included at seven different hospitals in Denmark and seen in outpatient clinics, including dedicated outreach clinics, to ensure that the patients who were most likely to be the target population were offered participation. Since the standard of care is well studied in other studies, we chose to randomize GLE/PIB + ribavirin for four weeks and GLE/PIB for eight weeks in the 2:1 ratio. Randomization was done after screening by the investigator and stratified by genotype 3/non 3 and handled by the research support organization Open Patient Data Explorative Network (OPEN) at the University of Southern Denmark [21], through a REDCap database. Randomization and stratification blocks was set up by an independent data manager from OPEN and blinded to the investigator. The study medicine was provided in weekly dosing boxes at treatment initiation and by treatment week two. Ribavirin was dosed as 15 mg/kg once daily with an upper limit of 1400 mg. In light of the results from phase 1, an interim analysis was planned when 10 patients had experienced virological relapse independent of the number of included patients.

### 2.2. Patients

We included patients age 18–49 with chronic hepatitis C, defined as two positive HCV tests with more than six months apart (at least one positive HCV RNA). All study participants had used intravenous drugs within the last year, including heroin-assisted treatment. An amendment in January 2020 changed this criterion to also include all patients with chronic hepatitis C who were treated with opioid agonist therapy (OAT), due to slow recruitment to the study. All genotypes were accepted and patients had to be treatment naïve and have an absence of any sign of liver fibrosis, defined as a liver stiff measurement (LSM) measured by Fibroscan less than 8 kPa. In addition, we excluded patients with cirrhosis, defined as clinical signs, International Normalized Ratio (INR) > 1.3 and platelets < 130 × 10^9^/L. Patients who were co-infected with hepatitis B (positive HBsAg), HIV (positive ag + ab) or had comorbidities with significant liver disease were excluded. Due to the use of ribavirin, patients with a hemoglobin level less than 7 mmol/L (11.3 g/dL), pregnancy or refusal to use contraceptives were excluded from the study.

### 2.3. 4RIBC Phase 1

**Figure 1:**
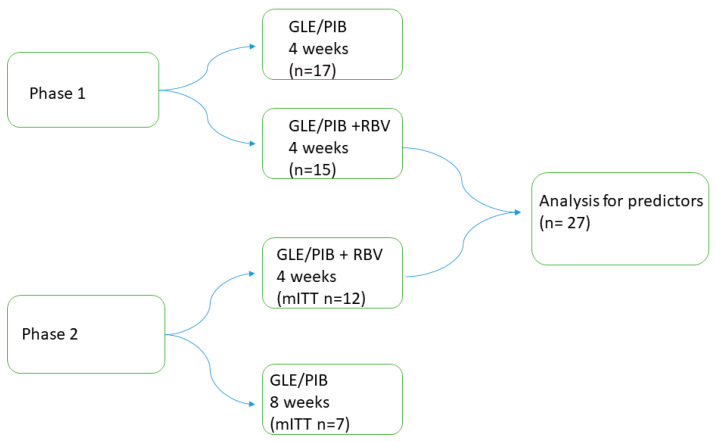
The 4RIBC study design of included patients. Abbreviations: glecaprevir/pibrentasvir (GLE/PIB); ribavirin (RBV); modified intention to treat (mITT).

Demographical-, clinical-, laboratory and outcome data from phase 1 were used in the analysis for predictors for achieving cure after four weeks’ treatment for chronic hepatitis C. Patients from phase 1 included in this analysis were all treated for four weeks with GLE/PIB with ribavirin, see Figure 1.

### 2.4. Data Collection

A REDCAP database served as the electronic case report form (e-CRF) where all clinical data were recorded. Initially patients were seen eight times during the 48-week study period with blood samples at each visit as in the first phase of the study [19]; see Supplemental Table S1. An amendment changed this in January 2020 so patients should only be physically present at the first three visits (screening, treatment start and two weeks after treatment start). Subsequent visits could be blood tests and telephone consultations at the discretion of the investigator. A blood sample at screening, post treatment week 12 and week 48 were compulsory. A dried blood spot (DBS) could replace blood samples at post treatment week 12 and 48. Infection time was defined as first year of injection of intravenous drugs.

### 2.5. Laboratory Methods

HCV RNA in plasma samples were either determined by the quantitative cobas^®^HCV assay (Roche Diagnostics GmbH, Mannheim, German) run at the cobas^®^

6800 system (Roche Molecular system) or by the Aptima HCV Quant Dx assay (Hologic GmbH, Vienna, Austria) run at the Panther^®^ system (Hologic, Marlborough, MA, USA). The lower quantification limit were 15 IU/mL and 10 IU/mL, respectively, and all analyses were performed according to the manufacturer’s instructions.

DBS were obtained on Whatman^®^ 903 protein saver cards (Sigma-Aldrich, Copenhagen, Denmark) and were allowed to dry for 1 to 3 days before they were eluted and tested for anti-HCV. HCV antibodies were determined by the Alinity *i* Anti-HCV assay (Abbott Diagnostics, Delkenheim, Germany). Anti-HCV positive samples were tested for the presence of HCV RNA by nucleic acid amplification testing using the cobas^®^ MPX assay (Roche Diagnostics GmbH, Mannheim, German) run at the cobas^®^ 6800 system (Roche Molecular system). The sensitivity when using DBS samples has previously been reported to be >95% [22]. 

### 2.6. Safety and Compliance

The investigator and a study nurse evaluated safety and compliance at each study visit until post treatment week 12. During treatment, all patients were asked for key symptoms related to ribavirin intake (headache, fatigue, nausea, dyspnea, rash, pruritus and diarrhea). In addition, spontaneously reported adverse events were reported and categorized into body systems using the Medical Dictionary for Regulatory Activities (MeDRA) classification of the WHO terminology. At the discretion of the investigator, all adverse events were graded in intensity from mild, moderate, severe to life threatening. Blood samples during treatment were, after an amendment in January 2020, only required if the patient reported clear, marked side effects, and especially when hemolytic anemia was suspected. Anemia was only reported as an adverse event if hemoglobin < 6.0 mmol/L (9.7 g/dL) was observed.

### 2.7. Virological Relapse

Measurable HCV RNA at SVR12 was defined as virological relapse. Analysis of resistance-associated substitutions (RAS) was performed by Sanger sequence. Patients who relapsed were offered retreatment with a sofosbuvir/velpatasvir (SOF/VEL) regime according to resistance analysis after treatment failure.

### 2.8. Statistic and Sample Size

The primary and all other secondary efficacy parameters were analyzed using the intent-to-treat population (ITT), including all patients who received at least one dose of study medicine. Lost to follow up was deemed to be a treatment failure. The modified intention-to-treat (mITT) population was defined to include all patients where final outcome (SVR12) was available. Sample size was based on a SVR12 rate of 91% in both arms with a power of 90%. This means that 130 patients needed to be randomized to GLE/PIB + ribavirin for four weeks and 65 to GLE/PIB for eight weeks to have a confidence level of 95% with a one-sided alpha at 0.05.

Descriptive statistics were reported as proportions for categorical variables and medians with interquartile ranges (IQR) for continuous variables. To test significance between groups we used Fisher’s exact test to compare categorical variables between groups and for continuously data we used the *t*-test for normally distributed data and the Wilcoxon Mann–Whitney test for non-normally distributed data. Continuously variables were transformed as appropriate. A *p*-value below <0.05 was considered significant. For data analysis to find predictors for cure, logistic regression was used to compute the odds ratio (OR) and 95% confidence intervals (CI). STATA version 15 (StataCorp LP, College Station, TX, USA) was used for data processing and analyses.

### 2.9. Ethical

Approval for this study was given by the Danish Health and Medicines Authority (EudraCT No. 2017-005179-21), The Regional Committee on Health Research Ethics for Southern Denmark (ID-S20180013) and the Danish Data Protection Agency (j.no 18/21965) and monitored by the Good Clinical Practice Institute at the University of Southern Denmark. All patients signed an informed consent and could withdraw their consent at any time during the study. Patients with virological relapse were withdrawn from the study and offered retreatment for chronic hepatitis C.

The study adheres to the CONCERT guidelines for reporting randomized clinical trials.

## 3. Results

**Figure 2:**
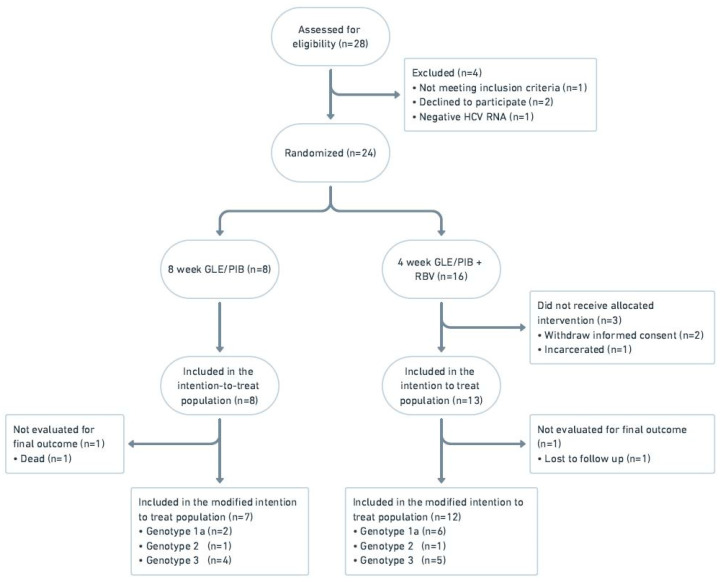
Flow diagram of the 4RIBC study, phase 2. Abbreviations: glecaprevir/pibrentasvir (GLE/PIB); ribavirin (RBV).

From March 2019 to February 2020, 28 patients were screened and included in the study, of whom 21 were included in the intention to treat population (see Figure 2). Patients were included at two of the seven centers participating in the study and followed according to protocol until August 2021.

In total 13 patients were randomized to four weeks’ treatment with GLE/PIB + ribavirin and eight patients received eight weeks’ treatment with GLE/PIB. Overall, one patient from each treatment arm was lost to follow up and were not evaluated for final outcome. The study was closed prematurely in September 2020 due to lack of recruitment of study participants.

### 3.1. Baseline Characteristics

Baseline characteristics for patients in the ITT group are shown in Table 1. All treated patients had reported current or former intravenous drug use and most patients were on OAT. The most common genotypes were 1 and 3. Interferon lambda 3 (INFL3) genotype CC was reported more frequently in the 8-week arm where the median baseline viral load was marginally lower and the median age lower. Overall, baseline demographic characteristics appeared well balanced between the treatment arms where the median infection duration for the two groups was about 20 years.

### 3.2. Virological Response

**Figure 3:**
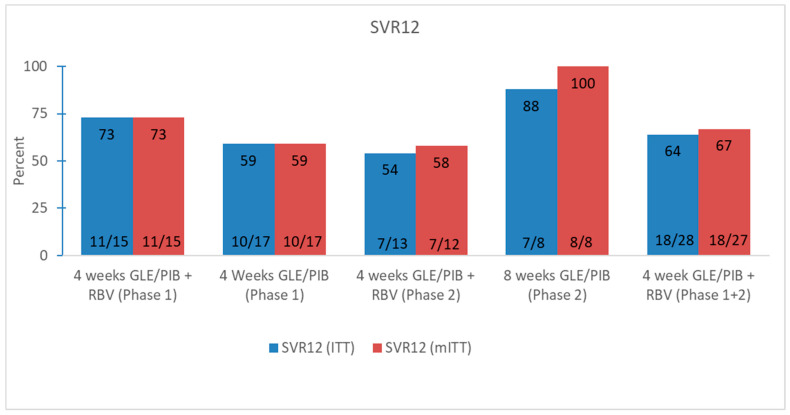
Virological responses for both phases of the 4RIBC study. Abbreviations: glecaprevir/pibrentasvir (GLE/PIB); ribavirin (RBV); sustained virological response week 12 (SVR12); intention to treat (ITT); modified intention to treat (mITT).

In the mITT group, 58.3% (7/12) (95% CI 0.28–0.85) achieved SVR12 if treated with GLE/PIB + ribavirin for four weeks and 100% (7/7) (95% CI 0.59–1) of patients who received GLE/PIB for eight weeks. In total, five patients experienced virological relapse, of whom one patient stated no risk behavior and high compliance during treatment and therefore was classified as a certain treatment failure. The other four patients with relapse had persistent risk behaviors for HCV infection during and after treatment and for two of them there were uncertainties about compliance during treatment. Sanger sequencing could not elucidate whether the virological relapse were reinfections or treatment failure. Three of the patients with virological failure had a baseline viral load above log_10_ 7.00 IU/mL. All five patients with virological relapse have since been retreated and cured with 12 weeks’ SOF/VEL. All patients who achieved SVR12, except two patients who were lost to follow, also obtained SVR48. By including the data from phase 1 the total SVR12 was 66.7% (18/27) (95% CI 0.46–0.83) for patients who were treated with GLE/PIB + ribavirin for four weeks (Figure 3).

### 3.3. Safety and Compliance

For the majority of patients, the treatment was well tolerated and adverse advents were mild and transient. Overall, 10 (76.9%) patients in the four-week treatment arm experienced at least one adverse advent whereas it was the case for eight patients (100%) in the eight-week arm. The most commonly reported adverse event was fatigue, which was more intensively reported for the patients who received ribavirin. One patient described side effects with suspected hemolytic anemia and blood samples revealed a hemoglobin decrease from 9.1 mmol/L (14.7 g/dL) to 6.3 mmol/L (10.2 g/dL) after 14 days of treatment with ribavirin. Ribavirin was reduced from 1400 mg to 600 mg and the hemoglobin was increased to the initial value, four weeks post treatment. The patient achieved SVR12. In addition, one patient who achieved SVR12 in the eight-week treatment arm stopped medicine intake after about 22 days due to gastrointestinal side effects. Overall, four severe adverse events were reported, none of which related to the intake of study drugs but all related to intravenous drug use including a death due to drug overdose.

For the ITT group a total of 74/784 (9%) missing doses were confirmed by simple pill count from returned dosing boxes and by patients’ own statement. In addition, two patients in the four-week treatment arm with virological relapse, did not return the dosing boxes and could not account for the number of taken tablets and their compliance to the treatment. The number of confirmed missed doses were highest for patients in the eight-week arm with an overall compliance of 83.5% to study drugs. Three patients had their treatment extended for three–seven days based on reported missed doses and one patient in the eight-week arm stopped treatment after about five weeks due to poor compliance.

### 3.4. Predictors for Achieving SVR12

**Figure 4:**
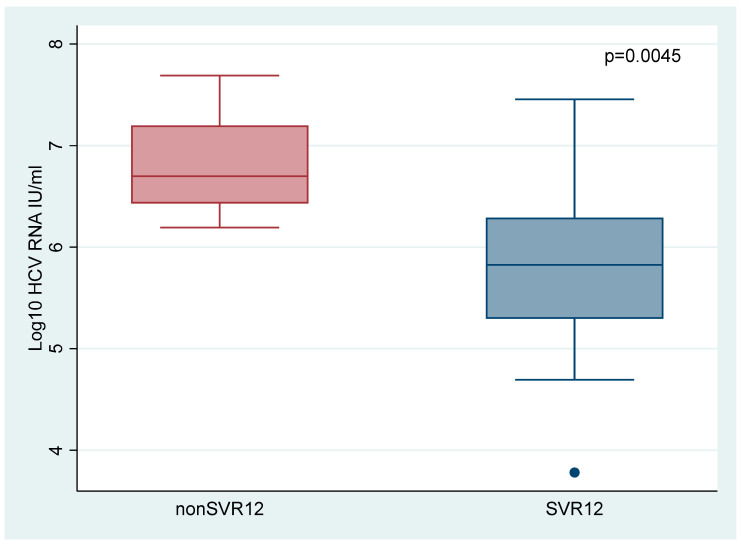
Baseline viral load according to SVR12 status. Abbreviations: sustained virological response (SVR).

Including the data from phase 1, a total of 27 patients received GLE/PIB + ribavirin for four weeks in the mITT group, of whom 18 (67%) achieved SVR12. Baseline characteristics for this population are shown in Supplemental Table S2. By comparing baseline characteristic for SVR12 patients and patients who experienced virological relapse it was revealed that low baseline viral load (*p* = 0.0045) (Figure 4) and genotype 3 (*p* = 0.042) were significant predictors for achieving cure (Table 2). Virological relapse was not observed in patients with a baseline viral load < 1,000,000 IU/mL, and among patients with a baseline HCV RNA < 2,000,000 IU/mL, 93.3% (14/15) of the patients achieved SVR12. Logistic regression showed an OR of 28 (95% CI 2.65–295.72, *p* = 0.006) for baseline viral load < 2,000,000 IU/mL and an OR of 10 (95% CI 1.03–97.50, *p* = 0.048) for genotype 3 in favor of achieving SVR12 (Supplemental Table S3). Overall, the baseline viral loads were lower for genotype 3 (mean log_10_ 5.9 IU/mL) than genotype non 3 (mean log_10_ 6.4 IU/mL). However, this correlation was not significant (*p* = 0.1673). No other significant predictors were found. Viral kinetics, defined as negative HCV RNA by treatment week two, was not associated with achieving SVR12 as six patients with positive HCV RNA after two weeks of treatment achieved SVR12.

## 4. Discussion

Although this study was discontinued prematurely, it is still one of the largest four-week treatment trials investigating second-generation, pan-genotypic DAA without a sofosbuvir containing regimen. By analyzing the overall results from the 4RIBC study, we found a SVR12 rate of 67% for patients treated with GLE/PIB + ribavirin for four weeks. The strongest predictors for cure were baseline viral load and genotype 3.

Despite the low overall cure rate for four weeks of treatment, we observed a 93% SVR12 among patients with a baseline HCV RNA < 2,000,000 IU/mL. Several other studies support that viral load is an important factor in reducing the treatment duration [14,18,23,24,25]. The reason why viral load was not included in this study design is that we wanted a simple and replicable design that easily could be transferred to clinical practice and especially in resource limited areas where preclinical baseline variables can be difficult to obtain. In addition, baseline viral load was not found to be a predictor of achieving SVR12 in the first phase of the study [19]. Another approach to reduce treatment duration is response-guided therapy. Here an on-treatment viral kinetic is used to individualize treatment duration without compromising the SVR12 rate [26,27]. In our study, we could not find any difference between measurable HCV RNA at treatment week two for patients who were cured and for those with virological relapse.

Other factors to consider in relation to viral kinetics is the choice of drug. DAA therapy causes a biphasic decline in HCV RNA with a first rapid decline and a second slower phase. The advantage of protease inhibitors (PI) is that they, in particular, induce a faster decline in viral load during the second phase and might more effectively restore the immune system to control intracellular HCV RNA [28,29]. Therefore, GLE/PIB was chosen as the DAA in this study. However, another trial investigating the combination of a NS5A, PI and NS5B (velpatasvir/voxilaprevir/sofosbuvir) found a SVR12 rate of only 27% after four weeks’ treatment [13].

In contrast to most other DAA studies, we found that patients with genotype 3 had an OR of 10 in favor of achieving SVR12. Genotype 3 has an overall lower response rate to DAA treatment, although this was only marginally lower in a recent GLE/PIB meta-analysis, 95.9 versus 98.1% SVR12 [30], and therefore genotype 3 patients have been omitted in most ultra-short treatment trials. Due to the very few patients included in our study these results should be interpreted with caution. However, these results suggest that genotype 3 patients also can be cured by short treatment and should not necessarily be excluded from future ultra-short treatment trials, especially if viral load is taken into account.

The hypothesis of this study was that by adding ribavirin to DAA the treatment duration could be reduced. Ribavirin has several important side effects, with the most important being hemolytic anemia [31]. In this study ribavirin was given for four weeks and not co-administrated with interferon. Both factors reduces the toxicity of ribavirin [32]. However, one patient in this study did develop hemolytic anemia after only two weeks of therapy with ribavirin. This demonstrates the disadvantage of including ribavirin, even in short treatment regiments.

An estimated 75% of people in need of hepatitis C therapy live in low- and middle-income countries (LMICs) [8,33]. Treatment has notably been scaled up in a few LMICs since 2015 but the majority of these countries are unlikely to reach the WHO goals by 2030 [8,34]. By reducing the treatment duration, a higher coverage can be reached within the same health budget. WHO now recommends pan-genotypic DAA with no need for staging of liver disease or pretreatment genotyping, which has limited the need for complex procedures and specialized laboratory services [8]. If short duration is to be a tool to enhance the treatment uptake, it must follow the same strategy. New advances with DBS entails that it is now possible to estimate the viral load by DBS [7] and therefore viral load can still, by simple methods, be implemented in short duration treatment programs.

SVR in short duration treatment may be dependent on genotypes and these differ by geographical region [35]. Patients of African origin are more often infected with unusual HCV genotypes 1 and 4 subtypes with impaired SVR12 rate [36,37]. In the first phase of the 4RIBC trial, we described such a patient of west-African origin and treatment failure after four weeks treatment with GLE/PIB. Full genome sequencing showed a novel subtype of the HCV genotype 1 [38]. Therefore, a standard short duration treatment may not fit all regions.

The cure rate after four weeks treatment with GLE/PIB + ribavirin from phase 1 was notably higher than the second phase of the trial, 73% versus 58%. The patient groups in the two phases differed substantially. Patients from phase 1 were included from the outpatient clinic affiliated to the hospital where they had been in routine control for years. The compliance in the first phase of the trial was high (99.8%) with only three missed doses distributed in three patients. Patients from phase 2 were included mainly from outreach clinics at drug treatment centers, and here the adherence to the study drugs was markedly lower, with 9.5% confirmed missed doses. Furthermore, we observed that the compliance rate was much lower in the eight-week arm than in the four-week treatment arm even though the SVR12 rate in the eight-week arm was 100% in the mITT population. This emphasizes that ultra-short treatment might be less forgiving than 8–12-week treatment courses, as a few missed doses may impede the patient’s chance of cure. Several studies have showed decreased adherence during treatment of eight–twelve weeks without any impact of the SVR12 rate [10,39].

Still, for vulnerable groups with low compliance, short duration treatment may be justified. Several studies in prison settings have shown this to be an effective way to scale up treatment for chronic hepatitis C [40], but prisoners with short sentences may not be offered treatment and for hospitalized patients, including psychiatric wards, the treatment duration will also be a critical factor for success.

Other areas where short treatment have been implemented with success are patients with recent infection [41] including to HCV negative recipients during kidney-, heart- and lung-transplantation from HCV-infected donor organs [42,43].

### Strengths and Limitations

The strength of the study is that the patients included (recent drug users) belong to the largest untreated group in many high-income countries where standard treatment uptake is poor. In addition, we included all genotypes thus making our results more applicable. The major limitation of this study is the small sample size. Patient recruitment was initially hampered by the inclusion requiring recent intravenous drug use, and after the inclusion criteria were broadened, the COVID-19 pandemic terminated the study prematurely. The small number of included patients reduced the power of the study to assess the true effect of ultra-short treatment with GLE/PIB + ribavirin, but with an SVR12 of 67%, it is unlikely that prolonging the inclusion phase would have led to an acceptable SVR12 (>90%).

## 5. Conclusions

The overall results from the 4RIBC study suggest that the majority of patients with favorable baseline characteristics, such as low viral load, can be treated for chronic hepatitis C with shorter treatment duration. Future studies are still needed to define the target population that may obtain the same cure rate as with standard of care.

## Figures and Tables

**Table 1:** Baseline Characteristics from phase 2 of the 4RIBC study for the intention to treat population.

Study Population, *n*	8 Weeks Glecaprevir/Pibrentasvir (*n* = 8)	4 Weeks Glecaprevir/Pibrentasvir + Ribavirin (*n* = 13)
Age (years) median (IQR)	36 (33.5–43.5)	42 (39–45)
Male, (%)	4 (50.0)	12 (92.3)
BMI, median (IQR)	23.0 (20.1–31.2) (*n* = 7)	23.8 (21.8–25.9) (*n* = 11)
Current or past alcohol overuse	5 (62.5)	8 (61.5)
Current or past intravenous drug use	8 (100)	13 (100)
HCV Genotype		
1a	2	6
2	1	1
3	5	6
HCV RNA (log_10_ IU/mL) median (IQR)	6.0 (4.4–6.9)	6.3 (6.0–6.6)
INFL3 genotype		
CC	5	3
Non CC	3	9
missing		1
LSM in kPa, median (range)	6.8 (4.1–7.7)	6.6 (4.1–7.5)
Opioid agonist therapy	7 (87.5)	11 (84.6)
Methadone	6 (75.0)	9 (69.2)
Buprenorphine	0 (0.0)	2 (15.4)
Heroin (legal)	5 (62.5)	3 (23.1)
Other	1 (12.5)	0 (0.0)
Year since infected, median (range)	20.5 (1–28)	21.5 (10–32) (*n* = 12)

Abbreviations: Interquartile range (IQR); Body Mass Index (BMI); Hepatitis C virus (HCV); Liver stiffness measurement (LSM); kilopascal (kPa); Interferon Lambda 3 (INFL3).

**Table 2:** Baseline characteristics in relation to SVR status for all patients in the mITT group for both phases of the 4RIBC study who received four weeks’ treatment with GLE/PIB + ribavirin.

	SVR (18)	Non SVR (9)	*p*-Value
Age, Median (IQR)	43 (39–46)	42 (40–44)	0.8195
Sex (Male/female)	13/5	7/2	1.000
Genotype 3/non3	10/8	1/8	0.042 *
INFL3 CC/non CC	5/13	1/7 *n* = 8	0.628
Weight in kilograms (IQR)	79.5 (72–89.5)	73 (69.2–82)	0.2365
OAT (IQR)	9 (50.0)	5 (55.6)	1.00
BMI, median (IQR)	26.4 (23.4–27.1) (*n* = 17)	24.9 (21.8–26.7) (*n* = 8)	0.4147
Viral load (log_10_ IU/mL) at treatment initiation, median (IQR)	5.8 (5.3–6.3)	6.7 (6.4–7.2)	0.0045 *
Current or past injection use (%)	16 (88.9)	8 (88.9)	1.000
Current or past alcohol use (%)	10 (55.6)	6 (66.7)	0.692
Years since infection, median (IQR)	21 (15–27) (*n* = 17)	23 (21–28)	0.1309
ALT (IU/L) at baseline median (IQR)	74.5 (44–112)	49 (44–60)	0.1107
Positive HCV RNA week 2 yes/no (%)	6/10 (37.5) (*n* = 16)	2/5 (28.6) (*n* = 7)	1.000

Abbreviations: Interquartile range (IQR); Body Mass Index (BMI); opioid agonist therapy (OAT); sustained virological response (SVR); alanine aminotransferase (ALT); * *p*-value below 0.05.

## Data Availability

Due to Danish rules on data availability, we are unable to make an anonymized dataset public. These rules are based on the Data Protection Act, imposed by The Danish Data Protection Agency. An English translation of the Data Protection Act can be found on the official website for The Danish Data Protection Agency (https://www.datatilsynet.dk/english/legislation/). For further information, the corresponding author can be contacted.

## Supplementary Materials

The following supporting information can be downloaded at: https://www.mdpi.com/article/10.3390/v14030614/s1, Table S1: Data Collection; Table S2: Baseline characteristics from phase 1 + 2 of the modified intention to treat population treated with four weeks of GLE/PIB + ribavirin; Table S3: Univariate analysis for predictors in favor of SVR12 after four weeks treatment with GLE/PIB + ribavirin.
