# Supplementary material for: Four Weeks Treatment with Glecaprevir/Pibrentasvir + Ribavirin—A Randomized Controlled Clinical Trial
Source: Viruses. 2022 Mar 16;14(3):614. doi: 10.3390/v14030614 (PMC8948928; doi:10.3390/v14030614)
Supplement: Supplementary file 1 [file viruses-14-00614-s001.zip › viruses-1640886-supplementary.pdf]

Supplemental Table S1 – Data Collection. Study procedure as outlines in tables below***.***

Table S1a:

**Assessments and procedures**	**Screening Week[h] -12-0**	**Start[h]**	**Day 14[h] (+/- 3 day)**	**Day 28[f](EOT for 4 weeks treatment) (+/- 2day)**	**Day 56[g] (EOT for 8 weeks treatment) (+/-2day)**	**Week 4 Post treatment (-1 /+14 days)**	**Week [12h,i] Post treatment (-1 /+14 days)**	**Week 24 Post treatment (-1 /+14 days)**	**Week [48h,i] Post treatment (-1 /+14 days)**
**Informed Consent**	x								
**Medical history**	x								
**Review of medication**	x	x	x	x	x	x	x		
**Physical Examination [a)]**	x	(x)	(x)	(x)	(x)	(x)	(x)	(x)	(x)
**Adverse events**		x	x	x	x	x	x		
**FibroScan[b)]**	x[a]								
**Height**	x								
**Weight[(c)]**	x	x	x	x	x	x	x	x	x
**ECG [b)]**	x								
**Vital signs [d)]**	x	x	x	x	x	x	x	x	x
**Questionnaires [e]**	x			x	x	x	x	x	x
**Compliance interview and pill count**			x	x	x				

a) Full examination as defined in the e-CRF at screening – afterwards only if symptoms present or as follow-up of existing findings
b) ECG and/or FibroScan less than 3 month old available in the subjects electronic or paper record accepted.
c) Includes abdominal circumference
d) Blood pressure, pulse
e) SF-36, the Fatigue severity scale (FSS) and the insomnia severity index(ISI)
f) Only for 4 weeks treatment
g) Only for 8 weeks treatment
h) Compulsory study visit.
i) Blood samples, as minimum is required.

Abbreviations: end of treatment (EOT)

Table S1b

Tests	Screening Week 12-0	Start[j]	Day 14[k]	Day 28[k] (EOT)	Day 56[k](EOT 8 weeks treatment)	Week 4 Post treatment	Week 12[l] Post treatment	Week 24 Post treatment	Week 48[l] Post treatment
**HCV-RNA**	X	x	x	x	x	x	x	x	x
**HCV genotype**	X					(x) if detectable	(x) if detectable	(x) if detectable	(x) if detectable
**Anti HAV IgM, HbsAg, antiHbc, Anti HIVa [a]**	X								
**IL 28b genotype [b]**	X								
**Haematology [c]**	X	x	x	x	x	x	x	x	x
**Renal function[d]**	X	x	x	x	x	x	x	x	x
**Liver function [e]**	X	x	x	x	x	x	x	x	x
**Immunoglobulins**	X								
**Pregnancy test[f]**	X	x					x	x	
**Thyroid function[g]**	X								
**Backup/archive[h]**	x (10ml)	x(10)ml	x(10ml)	x(15ml)[l]	x(15ml)	x(10ml)	x(10ml)	x(10ml)	x(10ml)
**Cell archive[i]**		x(10)ml	x(10)ml	x(10)ml	X(10ml)	x(10)ml	x(10)ml	x(10)ml	
**Approximate volume in milliliter blood Max total 400 ml**	55-70	45	55	60	60	45	50	50	35

a) Only if new risk behavior, or not documented within five years
b) Only if not documented in medical record
c) Hemoglobin, leucocytes , platelets,
d) Sodium, potassium, creatinin, estimated GFR, albumine
e) ALT, AST, bilirubin, GGT, ALP, INR, Gammaglutamyl-transferase, laktat dehydrogenase
f) All females – serum or urine HCG. Should be taken at treatment start and post treatment week 12 and 48.

g) *TSH (workup)*
h) *Archive sample will be separated and stored locally at -80°*
i) *Only for participants in phase 1*
j) *Will only be taken if it is more than 3 months since screening samples were taken.*

k) *Blood test can be omitted if no signs or suspicion of moderate or severe side effects.*
l) *Can be replaced by dried blood spot.*

Abbreviations: Hepatitis A virus (HAV), Hepatitis B surface antigen (HbsAg), antibody to Hepatitis B core antigen (antiHbc), antibodies to human immunodeficiency virus (anti HIV), glomerular filtration rate (GFR), alanine aminotransferase (ALT), Aspartate aminotransferase (AST), alkaline phosphatase (ALP), International normalized ratio (INR), human chorionic gonadotropin (HCG) , thyroid stimulating hormone (TSH)

Supplemental Table S2

Table S2: Baseline characteristics from phase 1 +2 of the total modified intention-to-treat-population for patients treated for 4 weeks with GLE/PIB + ribavirin.

**Study population, n**	**4 week**<br><br>**Glecaprevir/Pibrentasvir + Ribavirin (27)**
Age (years) median (IQR)	43 (39-45)
Male, (%)	20 (74.1)
BMI, median (IQR)	25.9 (23.1-27.1)<br><br>(n=25)
Current or past alcohol overuse, (%)	16 (59.3)
Current or past intravenous drug use	24 (88.9)
HCV Genotype	
1a	10
1b	2
2	3
3	11
4	1
HCV RNA (log_{10} IU/ml) median (IQR)	6.3 (5.6-6.8)
INFL 3 genotype	
CC	6

Non CC<br><br>Missing	20<br><br>1
LSM in kPa, median (range)	5.6 (4.6-6.9)
Opioid agonist therapy<br><br>Methadone<br><br>Buprenorphine<br><br>Heroin (legal)	14 (51.9)<br><br>11 (40.7)<br><br>3 (11.1)<br><br>3 (11.1)
Year since infected, median<br><br>(IQR)	22 (19-27)<br><br>(n=26)

Abbreviations: Interquartile range (IQR), Body Mass Index (BMI), Interferon Lambda 3 (INFL3)

Liver stiffness measurement (LSM),

Supplemental Table S3

Table S3. Univariate analysis for predictors in favor of SVR12 after 4 weeks treatment with GLE/PIB + ribavirin.

**Variables**	**Univariate**	
	**OR (95% CI)**	**p-value**
Age (years)	1.02 (0.87-1.19)	0.811
Age		
<= 35	1.6 (0.14-18.00)	0.704
>35	1 (ref)	
Sex		
Female	1.35 (0.21-8.82)	0.757
Male	1 (ref.)	
Genotype		
3	10 (1.03-97.50	0.048*
Non 3	1 (ref.)	
BMI, median (IQR)		
>25	1.26 (0.25-6.36)	0.782
<=25	1 (ref)	
Baseline viral load Log10	0.13 (0.03-0.69)	0.016*
Baseline viral load		
<=2000	28 (2.65-295.72)	0.006*
>2000	1 (ref)	
Baseline viral load		
<=3000	4.38 (0.78-24.47	0.093
>3000	1 (ref)	
Baseline ALT Log10	3.83 (0.67-21.83)	0.130
Current or past alcohol use	0.625 (0.12-3.32)	0.581
Current or past intravenous drug use	1.30 (0.08-12.76)	1.000
INFL3		
CC	2.69 (0.26-27.82)	0.406
Non CC	1 (ref)	

Infection time	0.893 (0.77-1.04)	0.138

Abbreviations: Odds ratio (OR), confidence interval (CI); Body Mass Index (BMI), alanine aminotransferase (ALT), Interferon Lambda 3 (INFL3). Statistically significant results marked with *
